# Innovation to Advance Cancer Control Equitably

**DOI:** 10.1200/GO.22.00301

**Published:** 2022-10-17

**Authors:** Tezer Kutluk, Sonali Johnson, Zuzanna Tittenbrun

**Affiliations:** 1 Hacettepe University Faculty of Medicine & Cancer Institute, Ankara, Turkey; 2 UICC, Union for International Cancer Control, Geneva, Switzerland

Over the past few decades, the world has witnessed major progress in cancer control with advances in technology, greater levels of investment in cancer programs, and innovation in the delivery of cancer care. However, as a leading cause of death, the cancer epidemic continues to touch people's lives and is a priority topic on the global health agenda. There is a large divide in access to cancer services both between and within countries, and it is critical that governments move forward with ways to improve equity in national cancer control policy and planning.

Innovation in cancer research is expanding the realm of what is possible in cancer prevention, early detection, diagnosis, treatment, and patient-centered care. At the same time, spiraling costs related to noncommunicable diseases (and cancer, in particular) mean that health authorities around the world need to assess and adopt technologies, policies, and practices that can deliver the greatest impact nationally. Although health systems need to show flexibility in fostering new and/or creative, cost-effective methods and technologies to improve cancer outcomes, innovation must bring benefit to the lives of patients, provide cost savings to the health system, and promote health equity to improve public health over the long term.

Innovation should be an integral part of health system strengthening. This does not only mean developing new capacities in technology, medicines, and diagnostics but also creative and new ways of thinking and doing in providing care to patients. For example, determinants of health system improvement include leadership, institutions, system design, technology,^1^ social innovation, and partnership with different stakeholders in the global health arena.^2,3^ Although cancer control became a priority on the global agenda after the 2011 high-level meeting of United Nations on the prevention and control of noncommunicable diseases, in recent years, the COVID-19 pandemic, conflicts around the world, humanitarian emergencies, and economic crises have had a significant impact on cancer control and broader global health policies.

The World Cancer Leaders' Summit (WCLS), a flagship event of UICC (Union for International Cancer Control) since its first edition in 2008, is a platform that brings together key decision makers from around the world and encourages debate on emerging issues related to cancer with the aim to shape the future of cancer control. This special series follows on from the WCLS held on October 25-26, 2021, under the theme of Driving innovation to advance cancer control equitably which identified many important facets of ensuring an enabling environment for innovation while promoting access to innovation for all. In the series, *JCO Global Oncology* and UICC, two key players in global cancer control, highlight the importance of innovation and, more specifically, the advancement of equity nationally, regionally, and globally. The series includes papers that examine what innovation means across the continuum of cancer control, how innovation can be assessed in different contexts, and what needs to be considered when implementing innovative policies, practices, and technologies from a health systems' perspective.

The journal collected eight papers from experts who are working on innovative projects to help close the equity gap in cancer care. The papers cover a variety of topics related to the use of new technologies such as digital health innovations to support a multidisciplinary team approach, molecular diagnostic technologies to improve pathology services, development of a counseling app to reduce the psychosocial impact of human papillomavirus (HPV) testing, and the use of genomics in pediatric brain tumors to transform outcomes in low- and middle-income countries (LMICs). It also includes partnership models such as a multinational partnership and collaboration on quality improvement for palliative care and a European network on inequalities. The series offers perspectives from different actors such as researchers in both high-income and resource-limited settings but also patient perspectives on ways to engage people living with cancer as drivers of change in equitable access to innovation. It also includes a perspective from the private sector on the value of partnership for health system strengthening and improving access to quality cancer care.

The commentary authored by Erfani et al underlines the significant gap in the use of molecular diagnostics between high-income countries and low-income countries, mainly because of affordability, technical capacity, and limited diagnostic and clinical infrastructure, where limited access to diagnosis is still a critical barrier to accessing cancer medicines. The paper points to the lessons learned from the use of molecular diagnostics in tuberculosis and other infectious diseases and discusses the need to close the global cancer pathology gap.^4^ The review in genomic innovation and pediatric brain tumors by Bailey et al discusses how genomic innovations have improved diagnosis, treatment stratification, and targeted therapies for brain tumors in children. The imbalance in the impact of genomic advances between high-income countries and LMICs needs to be addressed, and the authors offer suggestions in a resource appropriate stepwise fashion to benefit children with CNS tumors living in LMICs, including clinical trials, twinning and collaboration, and compassionate use programs.^5^ Israel^6^ in her commentary presents Novartis' breaking barrier challenge which commits to lower the system barriers that hinder access to treatment with a focus on three key areas—precision oncology, care integration, and data in action. These three studies focus on inequity in access to cancer care and suggest new ways to make progress in closing the care gap.

In their paper, Eaton et al presented the City Cancer Challenge (C/Can) initiative which leads a new way of working with cities to support local cancer solutions through a novel implementation framework to strengthen existing efforts in national cancer planning and drive a coordinated and systematic approach to cancer care in LMICs. A multidisciplinary team approach is characterized by four essential components including health care worker training, treatment guidelines, a decision-making process for complex cases, and auxiliary administrative support for auditing and feedback.^7^ Although the engagement of stakeholders is often discussed and seen as vital to implementing strong patient-centered cancer care, the participation of patients and their representatives in cancer policies is still absent or nascent in many LMICs. Samson et al underlined the lack of sufficient literature that addresses the level of involvement of cancer patient organizations in national cancer policies and highlighted challenges and opportunities to improve the representation of those organizations in policy dialogs in LMICs. The authors point to further investment in evidence-based capacity building to ensure that the inclusion of the voice of people living with cancer is a key criterion for the assessment of equitable access to cancer care.^8^ Focusing on the topic of palliative care in oncology, the quality improvement collaboration initiative organized between seven Indian health care organizations as mentee sites and US and Australian academic institutions as mentors, authored by Satija et al,^9^ discusses a promising model to foster shared learning and performance improvement.

Mobile health solutions offer the potential for improving patient-centered care. Antelo et al designed a counseling app to reduce the psychosocial impact of HPV testing on the basis of a user-centered design in a low- and middle-income setting. In their paper, they suggest that the app constitutes a low-cost, easy-to-use tool that can change how women access evidence-based information and counseling for HPV and support women's follow-up and adherence to treatment for cervical cancer.^10^

Finally, the European Cancer Organization highlights feasible solutions and steps targeting key inequalities in Europe related to social determinants such as age, sex, race and ethnicity, and east-west divide. The European Cancer Organization Inequalities Network has been working to elevate these issues on the EU agenda through the Europe's Beating Cancer Plan and the new Cancer Inequalities Register.^11^

What this series shows is that it is possible to take innovative approaches to scale taking on board principles of collaboration, participation, and empowerment. It is intended to serve as a reference for policy makers, program managers, clinicians, and cancer advocates to explore the potential implications of innovation to cancer management and control in international settings and to promote the vision of health for all.
